# Placenta Percreta Presents with Neoangiogenesis of Arteries with Von Willebrand Factor-Negative Endothelium

**DOI:** 10.1007/s43032-021-00763-4

**Published:** 2021-11-11

**Authors:** Alexander Schwickert, Wolfgang Henrich, Martin Vogel, Kerstin Melchior, Loreen Ehrlich, Matthias Ochs, Thorsten Braun

**Affiliations:** 1 Charité – Universitätsmedizin Berlin, corporate member of Freie Universität Berlin and Humboldt‐Universität zu Berlin, Department of Obstetrics, Berlin, Germany; 2 Charité – Universitätsmedizin Berlin, Corporate Member of Freie Universität Berlin and Humboldt-Universität zu Berlin, Department of Pathology, Pediatric Pathology and Placentology, Berlin, Germany; 3 Charité – Universitätsmedizin Berlin, Corporate Member of Freie Universität Berlin and Humboldt-Universität zu Berlin, Institute of Functional Anatomy, Berlin, Germany; 4 Charité – Universitätsmedizin Berlin, Corporate Member of Freie Universität Berlin and Humboldt-Universität zu Berlin, Department of Experimental Obstetrics, Berlin, Germany

**Keywords:** Placenta percreta, Placenta accreta spectrum, Abnormally invasive placenta, Neoangiogenesis, Immunohistochemistry, Von Willebrand factor

## Abstract

In placenta percreta cases, large vessels are present on the precrete surface area. As these vessels are not found in normal placentation, we examined their histological structure for features that might explain the pathogenesis of neoangiogenesis induced by placenta accreta spectrum disorders (PAS). In two patients with placenta percreta (FIGO grade 3a) of the anterior uterine wall, one strikingly large vessel of 2 cm length was excised. The samples were formalin fixed and paraffin-embedded. Gomori trichrome staining was used to evaluate the muscular layers and Weigert-Van Gieson staining for elastic fibers. Immunohistochemical staining of the vessel endothelium was performed for Von Willebrand factor (VWF), platelet endothelial cell adhesion molecule (CD31), Ephrin B2, and EPH receptor B4. The structure of the vessel walls appeared artery-like. The vessel of patient one further exhibited an unorderly muscular layer and a lack of elastic laminae, whereas these features appeared normal in the vessel of the other patient. The endothelium of both vessels stained VWF-negative and CD31-positive. In conclusion, this study showed VWF-negative vessel endothelia of epiplacental arteries in placenta accreta spectrum. VWF is known to regulate artery formation, as the absence of VWF has been shown to cause enhanced vascularization. Therefore, we suppose that PAS provokes increased vascularization through suppression of VWF. This process might be associated with the immature vessel architecture as found in one of the vessels and Ephrin B2 and EPH receptor B4 negativity of both artery-like vessels. The underlying pathomechanism needs to be evaluated in a greater set of patients.

**Supplementary Information:**

The online version contains supplementary material available at 10.1007/s43032-021-00763-4.

## Introduction

The proper placental development is a key precondition for a healthy human pregnancy. The trophoblast invades the uterine decidua and subsequently the uterine spiral arteries, causing the arteries to dilate, to ensure continuously an adequate blood supply to the growing fetus [1]. In placenta accreta spectrum (PAS), this process is enhanced: Invasive extravillous trophoblast cells in PAS exhibit a more mesenchymal phenotype than in normal placentation, even until the third trimester [2, 3]. Placental tissue can be seen invading through the myometrium and serosa, sometimes even into the urinary bladder, parametria, or even the colon [4–6]. In addition to excessive extravillous trophoblastic invasion, abnormal maternal vascular remodeling (i.e., neoangiogenesis) is one of the driving factors of increased placental invasiveness [7–10]. Angiogenic growth factors, such as vascular endothelial growth factor and angiopoietin-2, are upregulated in PAS lysates. Likewise, the expression of antiangiogenic proteins such as vascular endothelial growth factor receptor-2 (VEGFR-2), endothelial cell tyrosine kinase receptor (Tie-2), and soluble fms-like tyrosine kinase 1 (sflt-1) is reduced in syncytiotrophoblastic cells from PAS cases compared to normal placenta specimens [11]. In PAS, neoangiogenesis so far has been investigated on placental tissue samples. However, PAS is also characterized by large blood vessels that are present on the precrete surface area. Accordingly, subplacental hypervascularization is a well-known sign for placenta accreta spectrum in antenatal ultrasound scans [12, 13]. As these vessels are not found in normal placentation, they appear to be the result of PAS-induced vasculogenesis. The objective of this pilot study was to examine their histological structure for noteworthy features that might shed light on the pathogenesis of PAS-induced neoangiogenesis. Therefore, Gomori trichrome staining and Weigert-Van Gieson staining were used to assess the structure of the vessel walls and to determine whether they are veins or arteries. Additionally, to verify the histological classification of the vessels, immunohistochemical staining of the Eph family transmembrane ligand, Ephrin B2 (artery), and its receptor tyrosine kinase, EPH receptor B4 (vein), was performed [14, 15]. Finally, Von Willebrand factor (VWF) and platelet endothelial cell adhesion molecule (PECAM-1, CD31) were used to further characterize the endothelium of the vessels.

## Methods

### Sample Collection

Specimens were sampled from two patients at Charité University Hospital in Berlin with singleton pregnancies and placenta percreta of the anterior uterine wall FIGO grade of invasiveness 3a [16]. One strikingly large vessel (each of 2-cm length) was excised during caesarean section in both patients before the delivery of the fetus. Both women had no known internal diseases including Von Willebrand disease. They had provided signed informed consent for the examination of the samples under protocols approved by the Ethics Committees of Charité University Hospital in Berlin (No. EA1_031_15). The samples were formalin fixed immediately and paraffin-embedded for further analyses. 

### Patient Characteristics

**Fig. 1:**
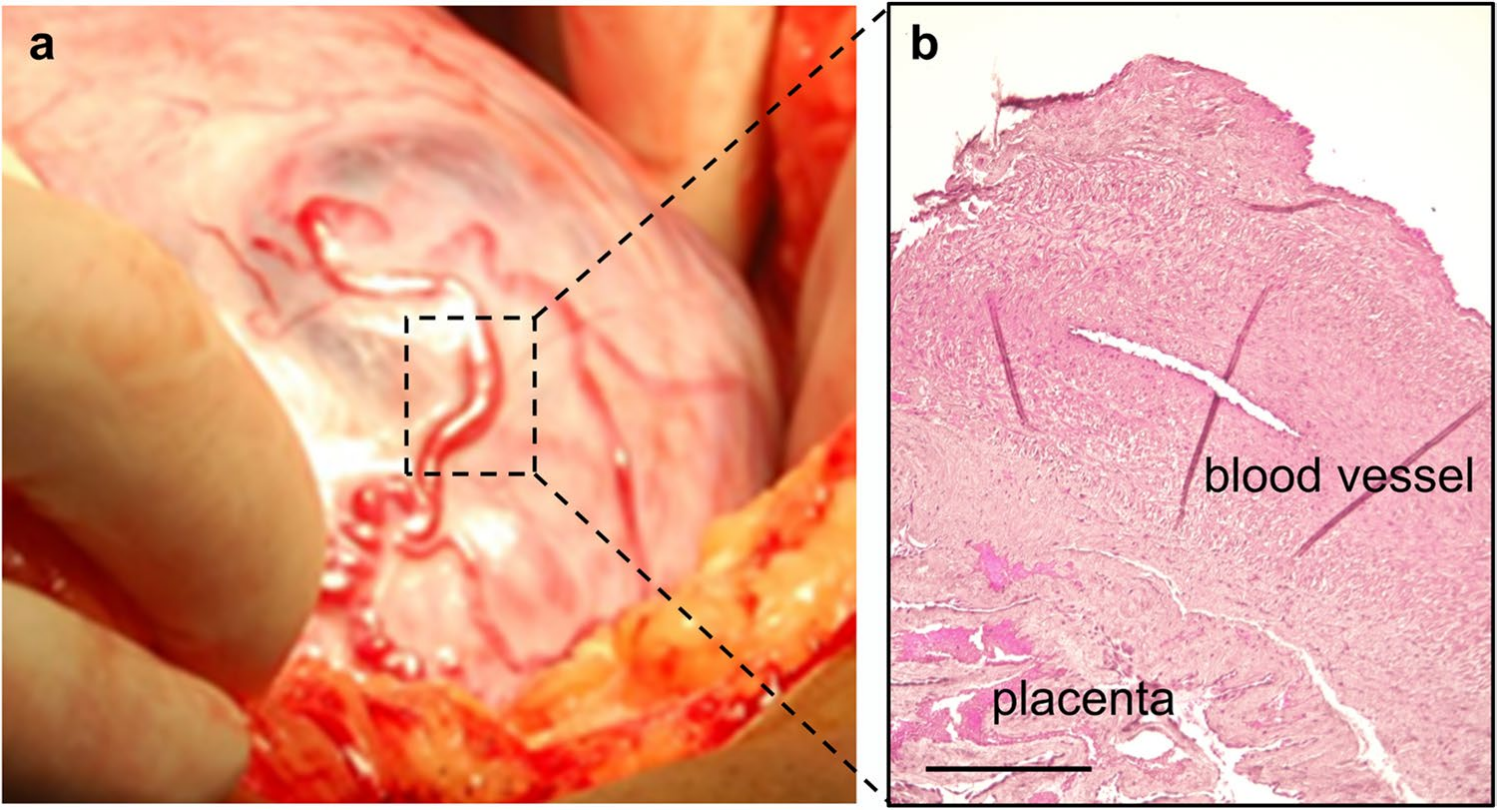
Placenta accreta spectrum (PAS) and neoangiogenesis. **a** Intraoperative image of the PAS area on the anterior wall of the uterus reveals big newly formed epiplacental vessels; the dashed box shows the sampling location; **b** sagittal vessel section with Weigert-Van Gieson staining. Scale bar = 0.5 mm

Patient 1 is a 33-year-old G2P1 with one previous emergency cesarean section due to fetal distress at term 2 years earlier. In this pregnancy, a planned cesarean section was performed at 35 weeks after placenta percreta had been suspected in antenatal ultrasound scans. Intraoperatively, anterior placenta previa percreta (FIGO grade of invasiveness 3a, Fig. 1) was seen, and subtotal hysterectomy was performed in consent with the patient and completed family planning with a perioperative blood loss of 900 ml and without further complications [16].

Patient 2 is a 39-year-old G3P2. She had one previous cesarean section at 35 weeks due to placenta previa six years before this pregnancy. At that time, the placenta had presented intraoperatively as accrete (FIGO grade of invasiveness 1) and had been treated through sutures in the uterine cavity, B-Lynch-sutures, and the insertion of an intrauterine balloon [16]. In this pregnancy, the placenta previa percreta had been suspected in antenatal ultrasound scans. Therefore, a planned cesarean section was performed at 35 weeks. Intraoperatively, anterior placenta previa percreta (FIGO grade of invasiveness 3a) was seen, and subtotal hysterectomy was performed in consent with the patient and completed family planning with a perioperative blood loss of 2500 ml and without further complications [16].

### Gomori Trichrome Staining

Paraffin Sects. 5-µm thick of the sagittal vessel were cut and dewaxed in xylene (2 × 5 min; J. T. Baker, Radnor, USA) and rehydrated in ethanol (2 min each in 96%, 70%, 50%; Carl Roth, Karlsruhe, Germany) followed by incubation for 30 min at 56 °C in Bouin solution. The sections were then rinsed under running water for 5 min. Incubation in Weigerts iron hematoxylin (Merck, Darmstadt, Germany) for 10 min followed by rinsing under running water for another 10 min. This was followed by incubation in trichrome solution (Sigma-Aldrich, Taufkirchen, Germany) for 25 min. Differentiation was performed in 0.5% acetic acid (Merck, Darmstadt, Germany) for 2 min and 1 min followed by dehydration in ethanol (1 min 96%, 2 × 2 min 100%) and xylene (2 × 5 min). The sections were covered with Entellan® Neu (Merck, Darmstadt, Germany) and a cover glass. As a result of the staining, cytoplasm and erythrocytes are shown in red, fibrin and muscle pink, nuclei blue to black, and collagen fibers green.

### Weigert-Van Gieson Staining

The procedure was carried out using the Elastica van Gieson staining kit (Merck, 1.15974.0002). About 5-µm-thick vessel paraffin sections were cut and deparaffinized in xylene (2 × 5 min; J. T. Baker, Radnor, USA) and rehydrated in ethanol (2 min each in 96%, 70%; Carl Roth, Karlsruhe, Germany) followed by incubation for 21 min in resorcinol-fuchsine solution. The sections were then rinsed under running water for 5 min and shortly rinsed with distilled water, followed by differentiation with 80% ethanol (immersed twice). Short rinse with distilled water. 3 min staining with Weigerts iron hematoxylin (Merck, Darmstadt, Germany), 10 min differentiation with running tap water, 3 min staining with van Gieson picrofuchsin solution. Short rinse in 70% ethanol, dehydration in ethanol (1 min 96%, 2 × 1 min 100%) and xylene (2 × 5 min). Covering with Entellan® Neu (Merck, Darmstadt, Germany) and a cover glass. As a result of the staining, elastic fibers are shown in black-violet, nuclei black blue/brown, collagen fibers red, and muscle cells/cytoplasm yellow.

### Immunohistochemical Staining of Endothelium Cells

Immunohistochemical staining was performed on 5-µm paraffin-embedded sections. The sections were deparaffinized and rehydrated. Heat induced or protease induced pre-treatment of deparaffinized sections was carried out followed by washing with phosphate buffered saline (PBS, pH 7.3) 3 times. Endogenous peroxidases were blocked. Samples were washed 3 times with PBS. Blocking of unspecific binding sites was performed followed by incubation with the primary antibodies monoclonal mouse anti-human CD31 (M0823, Agilent, Santa Clara, USA, diluted at 1:20), polyclonal rabbit anti-human VWF (A0082; Agilent, Agilent, Santa Clara, USA, diluted at 1:100), polyclonal rabbit anti-human EFNB2 (HPA008999; Sigma Life Science, St. Louis, USA, diluted at 1:20), and polyclonal goat anti-human EphB4 (AF3038; R&D Systems, Inc., Minneapolis, USA, diluted at 1:30). After rinsing 3 times with PBS, the tissue was incubated with the secondary antibody. After rinsing 3 times with PBS, samples were incubated in avidin–biotin-complex (PK-4002, Elite Vectastain ABC Kit, Vector Laboratories). The sections were twice washed with PBS and Tris hydrochloride, followed by DAB staining (3,3′-diaminobenzidin tetrahydrochloride, D-5637, Sigma-Aldrich, Taufkirchen, Germany) and washing with Tris hydrochloride, PBS, and water. Counterstaining of the cell nuclei was performed with hematoxylin staining (Weigert’s iron hematoxylin kit for nuclear staining in histology, 1,159,730,002, Sigma-Aldrich) followed by dehydration. The slides were covered under exclusion of air with Entellan® Neu (107,961, Merck, Darmstadt, Germany) under a cover glass. Negative controls were incubated without the primary antibody (Supplementary Fig. 1). All details on specific reagents and antibodies can be found in Appendix 1.

### Image Acquisition and Processing

Ten sections of every vessel were analyzed. Images of the tissue sections were acquired with the digital microscopy system Axioskop and the high-resolution digital color camera AxioCam MRc 5 (Zeiss, Oberkochen, Germany). Tissue sections were scanned at high magnification (× 100, × 200) and processed with Axiovision 4.8.2 software (Zeiss, Oberkochen, Germany).

## Results

Figure 1 shows the intraoperative photograph of the sampled blood vessel of patient one and Weigert-Van Gieson staining of the corresponding vessel. Images clearly demonstrate vessel localization on the surface of the percrete placenta.

### Structure of Vessel Walls

**Fig. 2:**
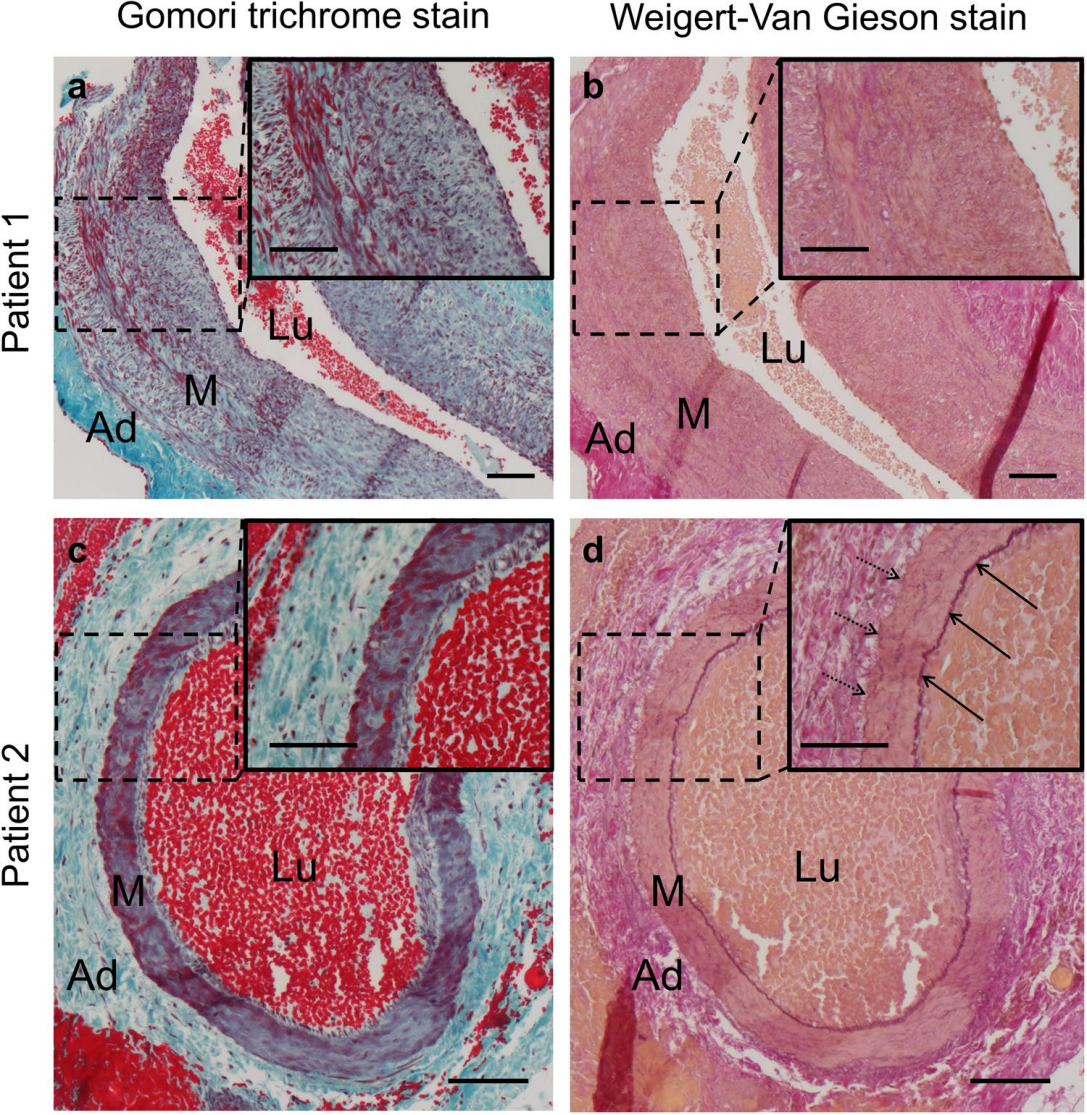
Gomori trichrome staining (**a,c**) and Weigert-Van Gieson staining (**b,d**) of sampled vessels. **a** Vessel of patient one with a thick muscular layer and **b** missing internal and external elastic laminae. **c,d** Vessel of patient two with thinner muscular layer and intact internal (arrows) and external elastic laminae (**d**, arrows with dotted line). As a result of the Gomori trichrome staining, cytoplasm and erythrocytes are shown in red, fibrin and muscle in pink, nuclei in blue to black, and collagen fibers in green. As a result of the Weigert-Van Gieson staining, elastic fibers are shown in black-violet, nuclei in black blue/brown, collagen fibers in red, and muscle cells/cytoplasm in yellow. Scale bars = 0.1 mm. *Ad* adventitia, *M* tunica media, *Lu* vessel lumen

**Fig. 3:**
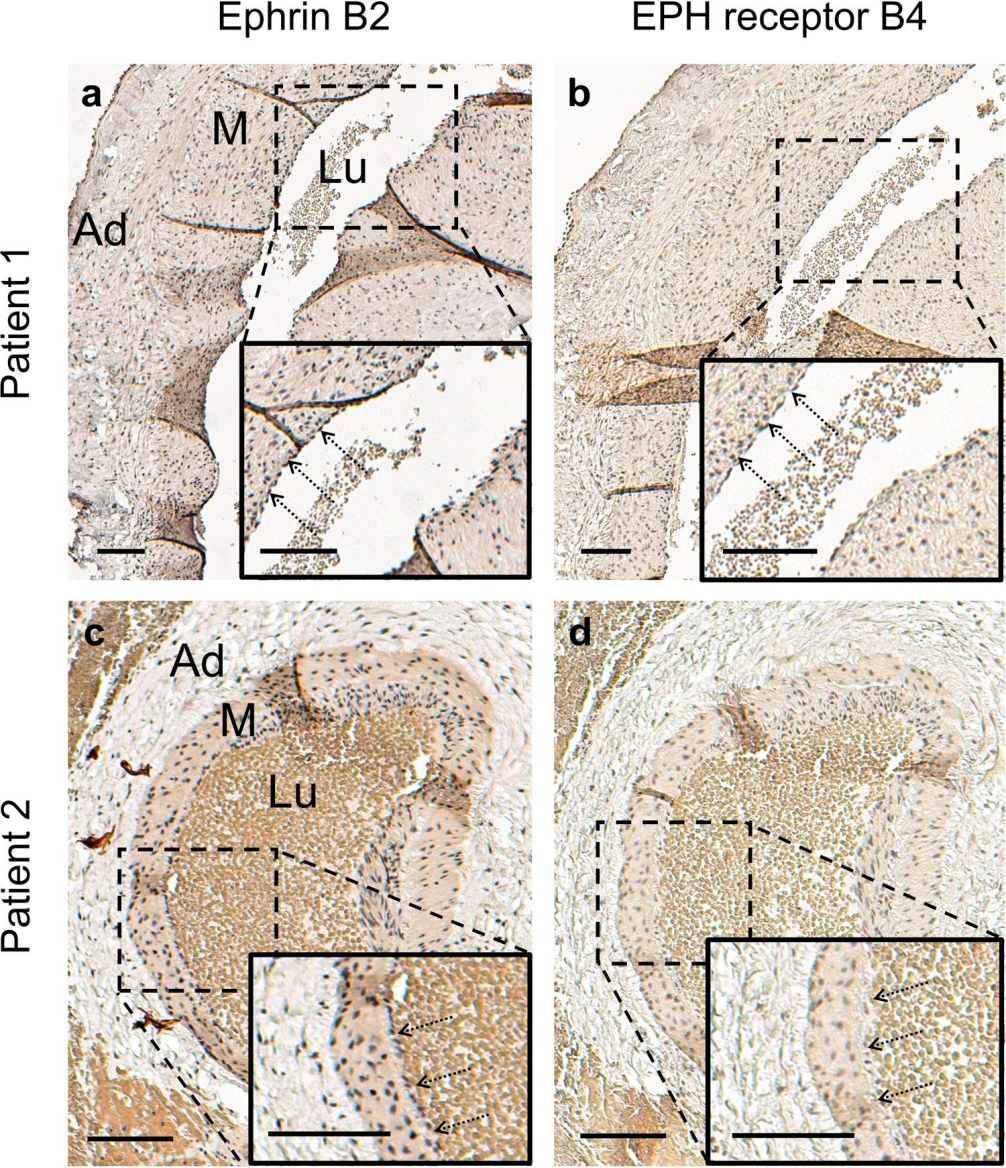
Immunohistochemical staining of Ephrin B2 and EPH receptor B4 of sampled vessels. The vessel endothelium of both patients stains negative for Ephrin B2 (**a,c**) and EPH receptor B4 (**b,d**). Arrows pointing at endothelium. Scale bars = 0.1 mm. *Ad* adventitia, *M* tunica media, *Lu* vessel lumen

Gomori trichrome staining showed that both vessels possess a clearly defined thick muscular layer and a vessel wall that appears not folded in the histological section, which characterized them most likely as arteries (Fig. 2). Vessel walls of both arteries showed a disorganized pattern of smooth muscle cells instead of a circular orientation as in other arteries [17]. Weigert-Van Gieson staining showed intact internal and external elastic laminae in the blood vessel of patient two (Fig. 2d), while elastic laminae were missing completely in the sample from patient one (Fig. 2b). Immunohistochemical staining of the endothelium for Ephrin B2 and EPH receptor B4 was negative in both vessels (Fig. 3).

### VWF-Negative Endothelium

**Fig. 4:**
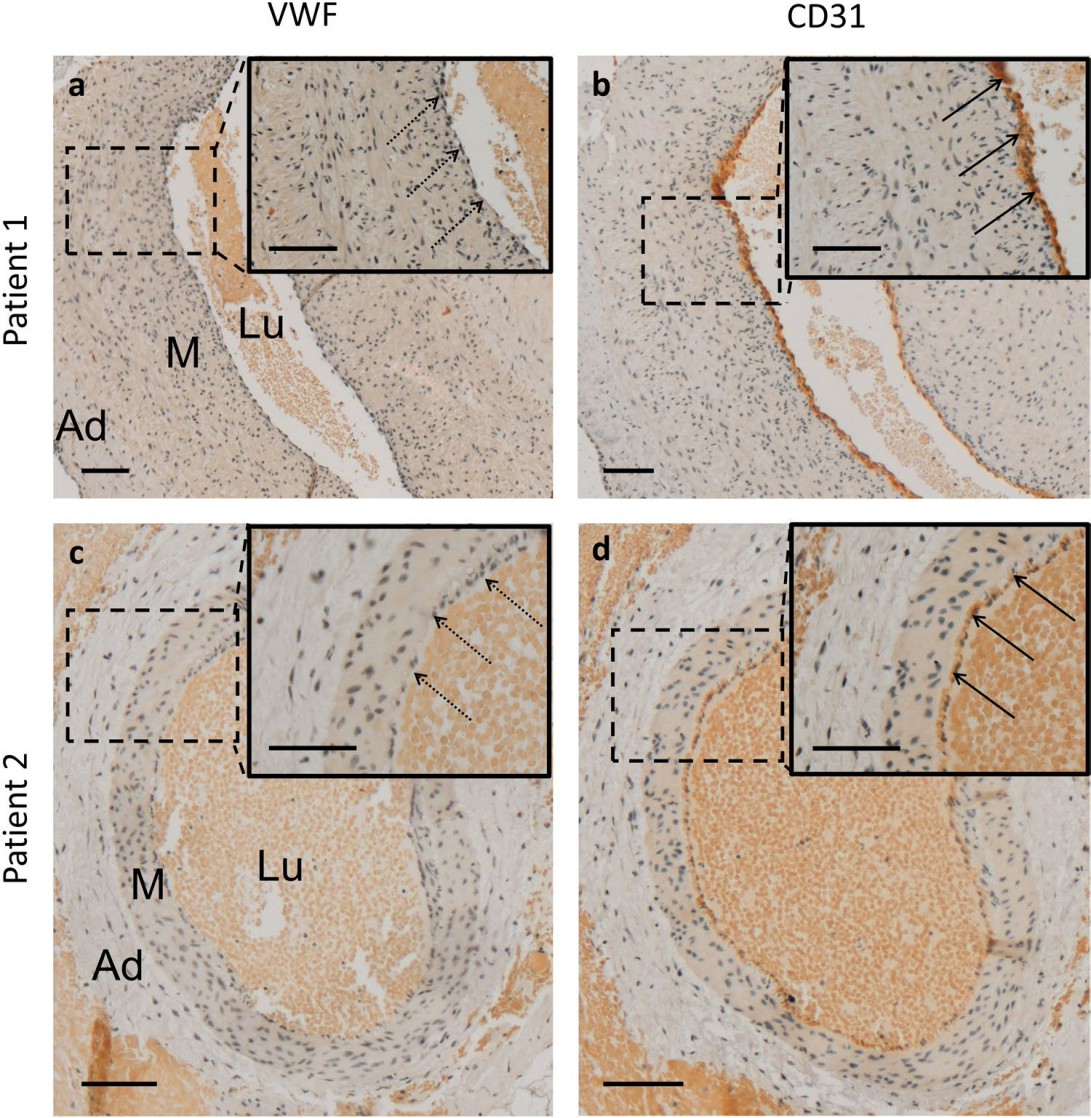
Immunohistochemical staining of Von Willebrand factor (VWF) and CD31 of sampled vessels. The vessel endothelium of both patients stains VWF-negative (**a,c**) and CD31-positive (**b,d**). Arrows pointing at endothelium. Scale bars = 0.1 mm. *Ad* adventitia, *M* tunica media, *Lu* vessel lumen

Immunohistochemical staining of VWF was negative in the endothelium of both patients’ arteries (Fig. 4a,c). However, both samples exhibited CD31-positive endothelial cells (Fig. 3b,d).

## Discussion

**Fig. 5:**
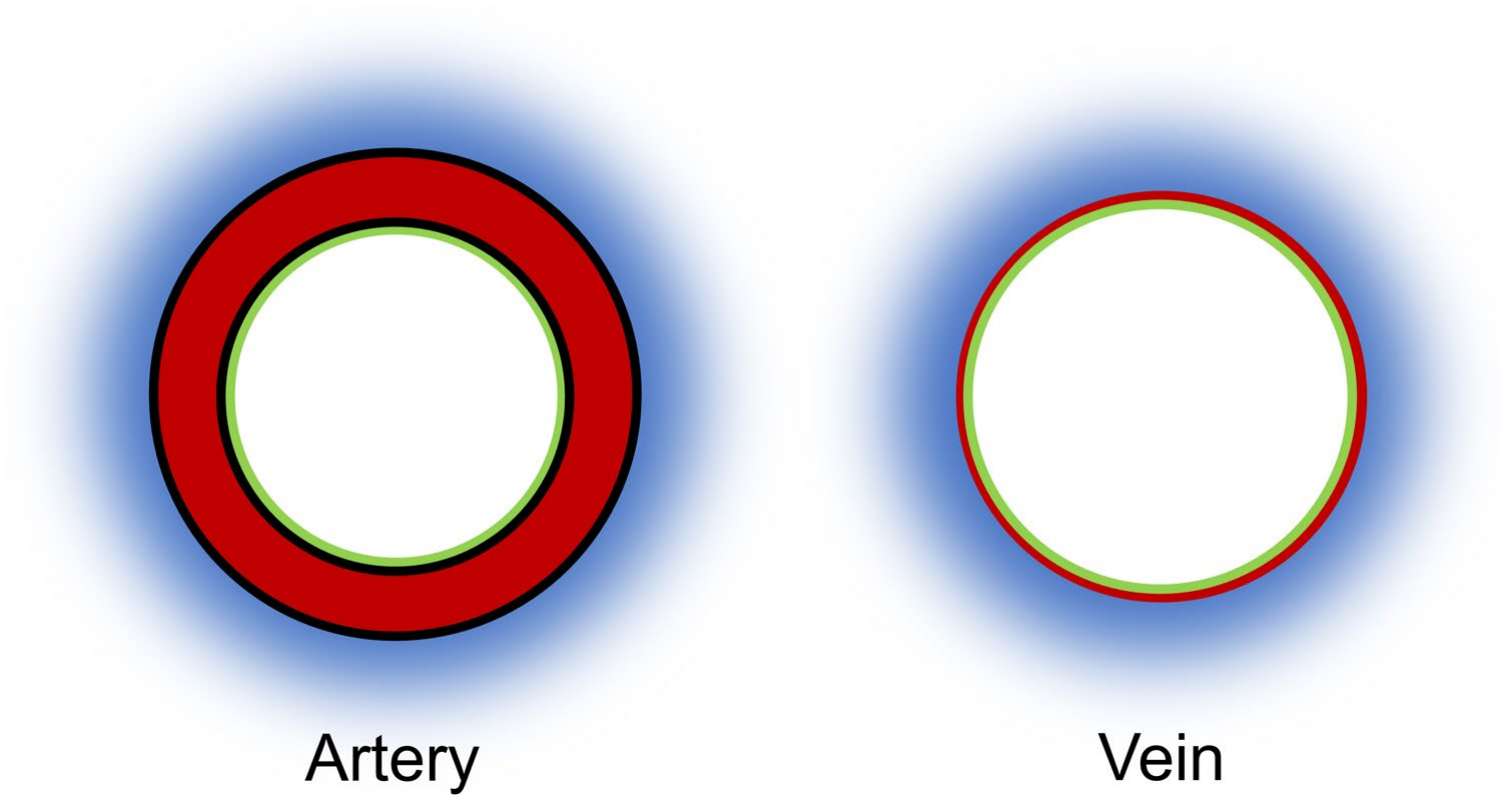
Schematic illustration of the wall structure of arteries and veins [17]. The wall of arteries is thicker than the wall of veins. In veins, the circular musculature is not as densely packed as in artery walls but is interspersed with abundant elastic and collagen fibers. Arteries have clearly visible internal and external elastic laminae. Green, endothelium; red, muscular layer; black, internal and external elastica laminae; blue, adventitia

Neoangiogenesis and abnormal maternal vascular remodeling are two driving forces of increased placental invasiveness [7, 8, 18]. This study describes two epiplacental blood vessels sampled from percrete placentae. Gomori trichrome staining showed that both vessels possess a clearly defined thick muscular layer and a vessel wall that appears not folded in the histological section, which characterized them most likely as arteries (Fig. 2, Fig. 5). Both vessels exhibit a VWF-negative endothelium. This is remarkable, as VWF has recently been shown to regulate artery formation [19]. It appears plausible that PAS induces neoangiogenesis by downregulation of VWF expression. This dysregulation of new vessel formation might explain the immature architecture with a disorganized muscular layer in both and a lack of elastic layers in one of the examined artery-like vessels. Several studies have shown that the absence of VWF causes enhanced vascularization in both in vitro and in vivo [20, 21]. In Von Willebrand disease, this phenomenon can lead to angiogenesis and angiodysplasia [22]. However, VWF has not yet been identified as a mediator of neoangiogenesis in PAS. Previous studies of PAS lysates have only described upregulation of angiogenic factors (vascular endothelial growth factor, angiopoietin, placental relaxin, relaxin family peptide receptor 1) and downregulation of antiangiogenic factors (vascular endothelial growth factor receptor 2, endothelial cell tyrosine kinase receptor 2, soluble fms-like tyrosine kinase 1) [7, 23]. Suppression of VWF might prove a further pathway that stimulates neoangiogenesis in PAS. It remains unclear, by which mechanism PAS modulates VWF expression. Placental hypoxia—due to an implantation of the placenta on a uterine scar—has been discussed as one factor that stimulates increased placental invasiveness [24–26]. It might also play a role in PAS-induced neoangiogenesis. Interestingly, though the blood vessels histologically showed an artery-like phenotype, the endothelium of both vessels stained negative for Ephrin B2, a unique molecular marker for arterial endothelial cells. A similar phenomenon has been shown in mouse embryos compound mutant for Foxc1 and Foxc2, two closely related Fox transcription factors [27]. The animals exhibit arteriovenous malformations and also lack induction of arterial markers such as Ephrin B2. The authors suggest that mutant endothelial cells “fail to acquire an arterial fate” [27]. We suspect that a similar mechanism—maybe associated with the lack of VWF—might be involved in neoangiogenesis in cases of PAS. The lack of elastic laminae found in one of the examined blood vessels can be explained by the fact that in PAS, extravillous trophoblast cells degrade elastic fibers within vessel walls through the expression of elastolytic proteases MMP-2, MMP-7, and MMP-9 [18, 28, 29]. A limitation of this pilot study stems from the fact that only specimens from two patients were examined.

## Conclusion

VWF is absent in the endothelial cells of the examined epiplacental blood vessels. The absence of VWF has been shown to induce neoangiogenesis and angiodysplasia. The endothelia of the examined artery-like vessels do not express Ephrin B2 and EPH receptor B4 – both unique molecular markers for arterial and venous endothelial cells, respectively. The next step is to evaluate the pathomechanism of this newly found phenomenon in PAS. This might eventually help in developing suitable treatment or even prevention strategies.

### Electronic supplementary material

Below is the link to the electronic supplementary material.

Supplementary Figure 1 Positive and negative controls of immunohistochemical staining of von Willebrand factor (VWF) (a, b), CD31 (c, d), Ephrin B2 (e, f) and EPH receptor B4 (g, h) of blood vessels in myometrial and placental samples. Arrows pointing at endothelium. Negative controls were incubated without the primary antibody. Scale bars = 0.1mm. Lu: vessel lumen (JPG 7984 KB)

## Data Availability

No statistical analyses were performed. All material is available upon request.

## Appendix 1

**Table 1:** Immunohistochemical staining protocols

Target antigen	Heat-induced pre-treatment	Blocking of endogenous peroxidases	Blocking of unspecific binding sites	Primary antibody	Secondary antibody	Incubation in avidin–biotin-complex	DAB staining
CD31	Dako Target Retrieval Solution (S1700, Dako, Agilent, Santa Clara, United States) for 20 min at 95 °C	3% Hydrogen peroxide for 20 min at room temperature	2% Normal horse serum (NHS, PK-4002, Elite Vectastain ABC Kit, Vector Laboratories, Germany) for 30 min	Monoclonal mouse anti-human CD31, number: M0823, Agilent, Santa Clara, USA, diluted at 1:20 in 2% NHS for 75 min	Biotinylated horse anti-mouse IgG, PK-4002, Vector Laboratories, Germany, diluted at 1:200 in 2% NHS for 30 min	30 min	30 min
Von Willebrand factor	Protease from Streptomyces griseus (P5147-5G, Sigma-Aldrich, Taufkirchen, Germany) for 5 min at 37 °C	3% Hydrogen peroxide and methanol for 20 min	2% Normal goat serum (NGS, S-1000, Vector Laboratories, Germany) for 60 min	Polyclonal rabbit anti-human VWF, number: A0082; Agilent, Agilent, Santa Clara, USA diluted at 1:100 in antibody dilution and incubated overnight at 4 °C	Biotinylated goat anti-rabbit, BA-1000, Vector Laboratories, Linaris GmbH, Wertheim-Bettingen, Germany, diluted at 1:500 in antibody dilution for 2 h	30 min	30 min
Ephrin B2	Target Retrieval Solution (10 x) (S1699, DakoCymation/Agilent Technologies Singapore Pte Ltd., Singapore) in a steamer for 10 min followed by 20 min at room temperature	3% Hydrogen peroxide and for 15 min	2% Normal goat serum (BA-1000/Vector Laboratories Inc., Burlingame, USA) for 90 min	Polyclonal rabbit anti-human EFNB2, HPA008999, Sigma Life Science, St. Louis, USA, diluted at 1:20 in PBS and incubated overnight at 4 °C	Biotinylated goat anti-rabbit, BA-1000, Vector Laboratories, Linaris GmbH, Wertheim-Bettingen, Germany, diluted at 1:200 in PBS for 2 h	120 min	15 min
EPH receptor B4	Target Retrieval Solution (10x) (S1699, DakoCymation/Agilent Technologies Singapore Pte Ltd., Singapore) in a water bath at 95 °C for 20 min followed by 20 min at room temperature	3% Hydrogen peroxide and for 15 min	5% Milk powder in PBS for 30 min	Polyclonal goat anti-human EphB4, number: AF3038; R&D Systems, Inc., Minneapolis, USA, diluted at 1:30 in PBS and incubated overnight at 4 °C	Polyclonal donkey anti-goat VisUcyte HRP Polymer Antibody (VC004, R&D Systems, Inc., Minneapolis, USA) diluted at 1:300 in PBS for 2 h	60 min	15 min

All non-specified incubations were performed at room temperature.
