# Supplementary figures and images for: Placenta Percreta Presents with Neoangiogenesis of Arteries with Von Willebrand Factor-Negative Endothelium
Source: Reprod Sci. 2021 Nov 11;29(4):1136–44. doi: 10.1007/s43032-021-00763-4 (PMC8907099; doi:10.1007/s43032-021-00763-4)

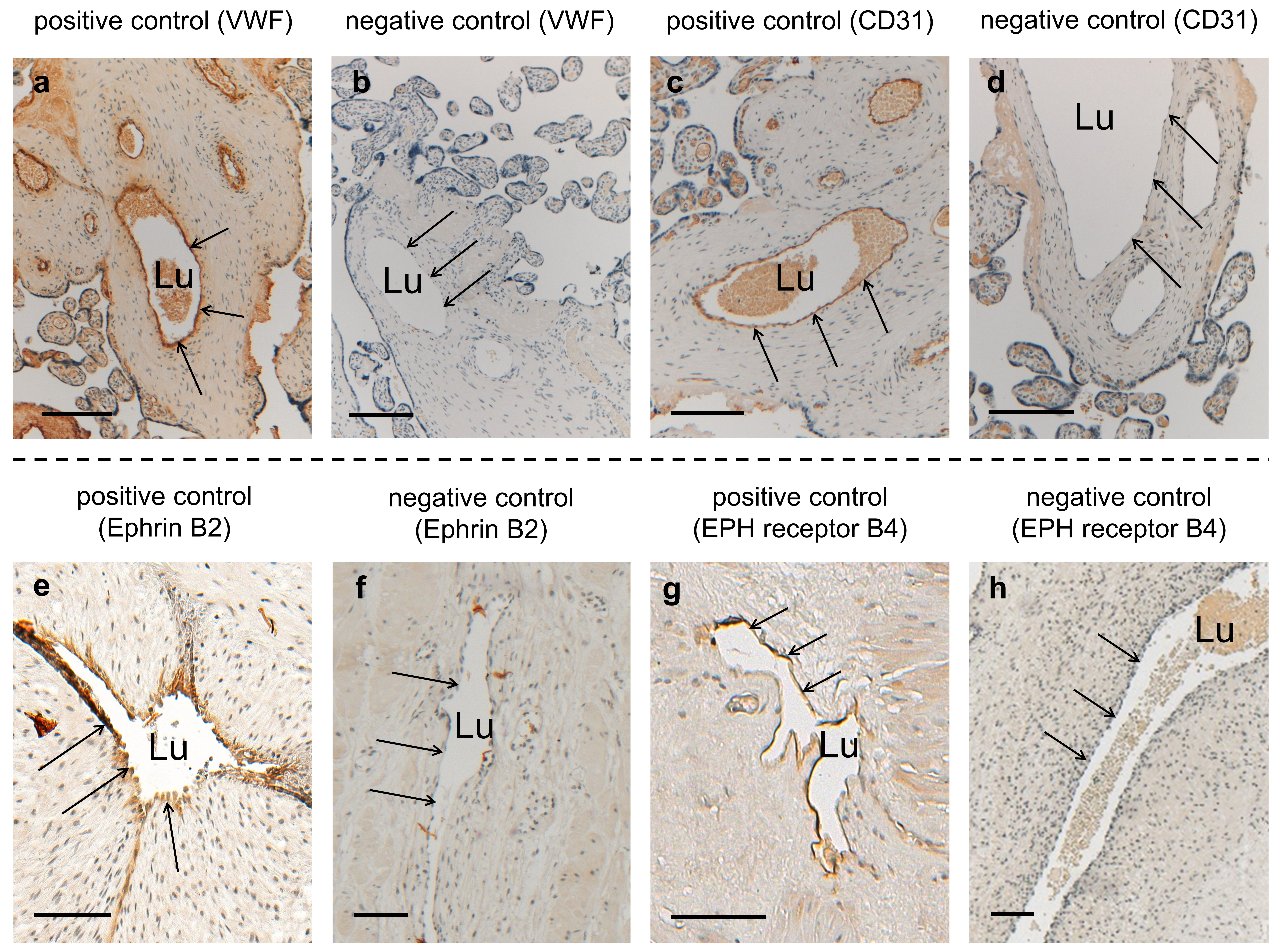

Supplement: Supplementary file 1 — Positive and negative controls of immunohistochemical staining of von Willebrand factor (VWF) (a, b), CD31 (c, d), Ephrin B2 (e, f) and EPH receptor B4 (g, h) of blood vessels in myometrial and placental samples. Arrows pointing at endothelium. Negative controls were incubated without the primary antibody. Scale bars = 0.1mm. Lu: vessel lumen (JPG 7984 KB) [file 43032_2021_763_MOESM1_ESM.jpg]
